# Identification of reliable reference genes for qRT-PCR studies of the developing mouse mammary gland

**DOI:** 10.1038/srep35595

**Published:** 2016-10-18

**Authors:** Anoeska Agatha Alida van de Moosdijk, Renée van Amerongen

**Affiliations:** 1 Section of Molecular Cytology and Van Leeuwenhoek Centre for Advanced Microscopy, Swammerdam Institute for Life Sciences, University of Amsterdam, Science Park 904, 1098 XH Amsterdam, The Netherlands

## Abstract

Cell growth and differentiation are often driven by subtle changes in gene expression. Many challenges still exist in detecting these changes, particularly in the context of a complex, developing tissue. Quantitative reverse transcription polymerase chain reaction (qRT-PCR) allows relatively high-throughput evaluation of multiple genes and developmental time points. Proper quantification of gene expression levels by qRT-PCR requires normalization to one or more reference genes. Traditionally, these genes have been selected based on their presumed “housekeeping” function, with the implicit assumption that they are stably expressed over the entire experimental set. However, this is rarely tested empirically. Here we describe the identification of novel reference genes for the mouse mammary gland based on their stable expression in published microarray datasets. We compared eight novel candidate reference genes (*Arpc3, Clock, Ctbp1, Phf7, Prdx1, Sugp2, Taf11* and *Usp7)* to eight traditional ones (*18S, Actb, Gapdh*, *Hmbs*, *Hprt*, *Rpl13a*, *Sdha* and *Tbp*) and analysed all genes for stable expression in the mouse mammary gland from pre-puberty to adulthood using four different algorithms (GeNorm, DeltaCt, BestKeeper and NormFinder). *Prdx1, Phf7* and *Ctbp1* were validated as novel and reliable, tissue-specific reference genes that outperform traditional reference genes in qRT-PCR studies of postnatal mammary gland development.

One of the challenges in developmental biology is to understand how subtle changes in gene expression drive growth and differentiation of complex tissues. The mouse mammary gland serves as a prime example of a dynamic and complex tissue that harbours many different cell types. During embryonic development, the mammary placode develops into a rudimentary mammary epithelial tree^1^,^2^. After birth, this epithelial tree remains relatively quiescent until the onset of puberty, when the epithelium grows out via branching morphogenesis and invades the surrounding fat pad. By the time the mouse reaches adulthood, the ducts have reached the end of the fat pad. However, dynamic growth and differentiation do not stop at this stage. Adult mice undergo a complete estrus cycle every 4–5 days, and during this time the epithelium also expands and regresses. Furthermore, during pregnancy the gland forms large alveolar structures that will produce milk during lactation once fully differentiated. After lactation, the mammary gland involutes and is reshaped back to a virgin-like state. This cycle can repeat itself multiple times throughout the reproductive life of an animal. As a result, during postnatal development the mammary gland does not only dramatically change in size, but also in cell type composition and differentiation status^3^,^4^.

Quantitative reverse transcription polymerase chain reaction (qRT-PCR) is a highly sensitive method for measuring changes in gene expression across multiple experimental conditions. However, even with current technologies it remains challenging to compare different developmental timepoints^5^,^6^,^7^,^8^,^9^,^10^,^11^, due to changes in tissue composition. To control for technical errors, individual samples are typically normalized against the expression of one or more internal reference genes. The ideal reference gene shows stable expression across the entire experimental dataset and is not influenced by any of the experimental parameters. This ensures that changes in reference gene expression are only due to technical variation such as pipetting errors and differences in sample input. When analysing genes that show subtle expression differences, as is the case for many developmental regulators, even small changes in reference gene expression can lead to aberrant results. It is therefore important to use the most stably expressed reference possible. Including more than one reference gene for the analyses further minimizes the effect of individual reference gene expression variation. While not yet commonplace, the use of at least three *bona-fide* reference genes is advised for proper normalization^12^.

Reference genes have historically been picked based on their presumed “housekeeping” function, ensuring that they would be abundantly expressed and easily detectable. However, traditionally used reference genes such as *Gapdh, 18S* and *Actb* can change expression in response to experimental treatment and might therefore not be appropriate to use under all circumstances^13^. Attempts have been made to identify universal reference genes, which could be applied to any sample of interest irrespective of its developmental origin, by comparing published datasets of multiple different human tissues and cell lines^14^,^15^,^16^,^17^,^18^. However, so far this has failed to yield a consistent list of candidate genes, raising the question whether such universal references exist at all^19^. Therefore, finding the best performing reference genes requires a dedicated effort focussing on the specific tissue or organism of interest^5^,^7^,^20^,^21^,^22^,^23^,^24^.

Because of the dynamic growth and differentiation properties of the mammary gland, it is particularly challenging to find genes that can serve as reliable reference genes across different stages of postnatal development. In 2010, Han and colleagues ranked the stability of eight commonly used reference genes for qRT-PCR studies of the mouse mammary gland^25^. However, their study focussed on the pregnancy and lactation stages in the adult only. Moreover, a single algorithm was used to rank the genes, while several algorithms are currently available to provide a more comprehensive overview of reference gene stability and sensitivity^12^,^26^,^27^,^28^,^29^.

Here we describe the identification of novel candidate reference genes for the mouse mammary gland based on their stable expression in multiple microarray datasets. We validate *Ctbp1*, *Phf7* and *Prdx1* as tissue-specific reference genes. They show stable expression across multiple stages of postnatal mammary gland development and allow the detection of subtle changes in *Wnt* gene expression in whole mammary gland RNA preparations. As such, they outperform traditionally used reference genes and are exquisitely suited for comparative qRT-PCR analyses in this complex developing tissue.

## Results

### Selection of novel candidate reference genes from published microarray data

**Figure 1:**
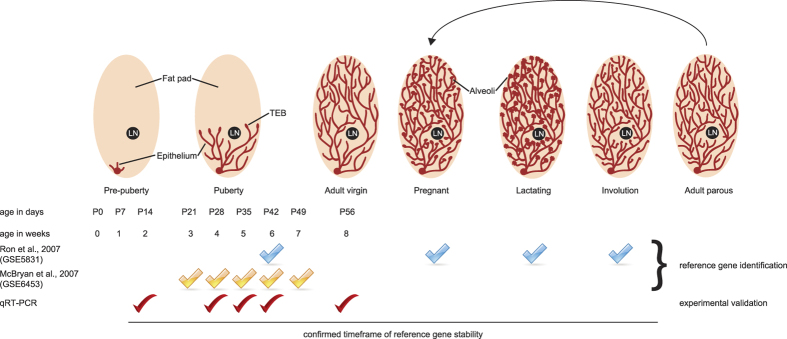
Postnatal development of the mouse mammary gland. Top: scheme illustrating developmental dynamics of the mammary gland. Before puberty, the fat pad only contains a rudimentary epithelial tree that starts from the nipple. During puberty, the epithelial tree invades the surrounding fat pad. This process is driven by highly proliferative structures called terminal end buds (TEBs). At the start of adulthood, the epithelial tree has fully invaded the fat pad and the TEBs regress. During each pregnancy, the gland forms alveoli for milk production during lactation. After weaning of the pups, the epithelial tree reverts back to a virgin-like state in a process called involution. LN = lymph node. Bottom: overview of the experimental time points used to generate the microarray expression data sets GSE5831 and GSE6453, as well as the time points used for qRT-PCR validation experiments.

The mammary gland is composed of multiple different cell types, including basal and luminal epithelial cells, stromal fibroblasts and adipocytes, as well as cells contributing to other structures in the gland such as nerves and blood vessels. In addition, the gland shows dynamic growth and differentiation during puberty, pregnancy and lactation, as well as extensive tissue remodelling during involution (Fig. 1). To date, no dedicated attempt has been made to determine whether suitable reference genes exist for use in qRT-PCR studies of whole mammary gland preparations across all stages of postnatal development. Therefore, we set out to identify novel, tissue-specific candidate reference genes that are stably expressed from pre-puberty through puberty and adulthood by analyzing published expression datasets.

We identified two gene expression profiling studies in the NCBI gene expression omnibus (GEO, http://www.ncbi.nlm.nih.gov/geo/) that compared different stages of mammary gland development (Fig. 1). Both datasets (GSE5831^30^ and GSE6453^31^) used microarrays to analyse the whole mammary gland transcriptome, but the studies were performed by different labs, on different mouse strains (C57BL6/J and CD-1, respectively) and using different array platforms. Furthermore, one experiment (GSE5831) compared adult stages (6 week old virgins, pregnancy day 14, lactation day 10 and involution day 4), whereas the other (GSE6453) compared pubertal time points (3, 4, 5, 6 and 7 week old females). We hypothesized that any gene that would be stably expressed across all of these arrays could qualify as a suitable reference gene. To identify these genes, we compared physiologically divergent stages using GEO2R, an interactive webtool that allows gene expression analysis of published microarray datasets using the GEOquery and limma R packages^32^.

**Figure 2:**
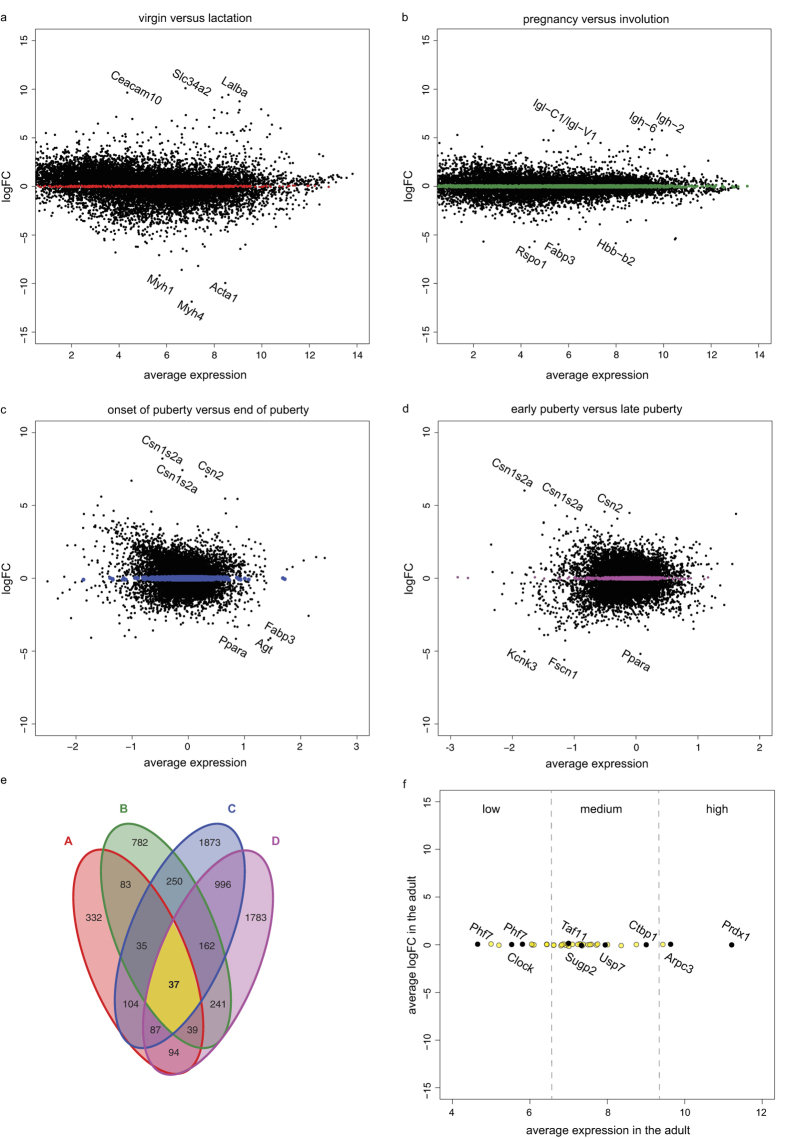
Identification of novel candidate reference genes. (**a–d**) MA plots for GSE5831 (**a,b**) and GSE6453 (**c,d**), comparing the virgin versus the lactating gland (**a**), the pregnant versus the involuting gland (**b**), the onset versus the end of puberty (3 versus 7 weeks, **c**) and early versus late puberty (4 weeks versus 6 weeks, **d**). The top 3 up- and downregulated genes for each of the four comparisons are indicated. Genes with a log fold change (logFC) between −0.1 and 0.1 are highlighted. (**e**) Venn diagram showing the overlap of genes with a logFC between −0.1 and 0.1 identified in **a–d**. Only 37 genes are shared between all four comparisons. (**f**) Ranking of the 37 genes with a logFC between −0.1 and 0.1 according to their average expression level in the GSE5831 dataset. The names of the candidate reference genes that were picked for further testing are highlighted.

First, we performed differential gene expression analysis of GSE5831 for the virgin and lactating, terminally differentiated gland. We selected genes with a log fold change (logFC) between −0.1 and 0.1, thus reflecting stable expression between the two conditions (Fig. 2a). Next, we performed a similar analysis for the pregnant (actively growing) and involuting (regressing) gland (Fig. 2b). Of the 852 and 1754 genes that showed stable expression in the virgin/lactating and pregnant/involuting comparisons, respectively, only a subset (194 genes) approached a logFC of 0 in both.

To narrow down the number of potential candidate reference genes, we then performed differential gene expression analysis of GSE6453 for different pubertal stages, comparing the start and end of puberty (3-week versus 7-week old mice, Fig. 2c) as well as early and late puberty (4-week versus 6-week old mice, Fig. 2d). This resulted in a list of 37 genes that showed stable expression across all of the tested conditions (Fig. 2e and Supplementary Table S1). From this list, we selected a final set of 8 putative reference genes (*Arpc3*, *Clock*, *Ctbp1*, *Phf7*, *Prdx1*, *Sugp2*, *Taf11* and *Usp7*, Fig. 2f). We made certain that the resulting candidates represented a variety of expression levels (Fig. 2f) and divergent functions as predicted by gene ontology analysis (Supplementary Table S1), and ensured that gene-specific qRT-PCR primers with unique, intron-spanning sequences and sufficient amplification efficiency could be designed (Supplementary Table S2).

### Variation in expression levels of candidate reference genes

Previously, Han and colleagues investigated the stability of eight commonly used reference genes in the pregnant and lactating mouse mammary gland^25^. To determine whether any of these genes are also stably expressed over a larger developmental timeframe, we selected four of the most stable genes identified in this study (*Rpl13a, Hprt, Gapdh* and *Actb*) as well as four additional commonly used reference genes^33^,^34^,^35^ (*18S, Hmbs, Tbp* and *Sdha*) as a “traditional reference gene set”.

**Figure 3:**
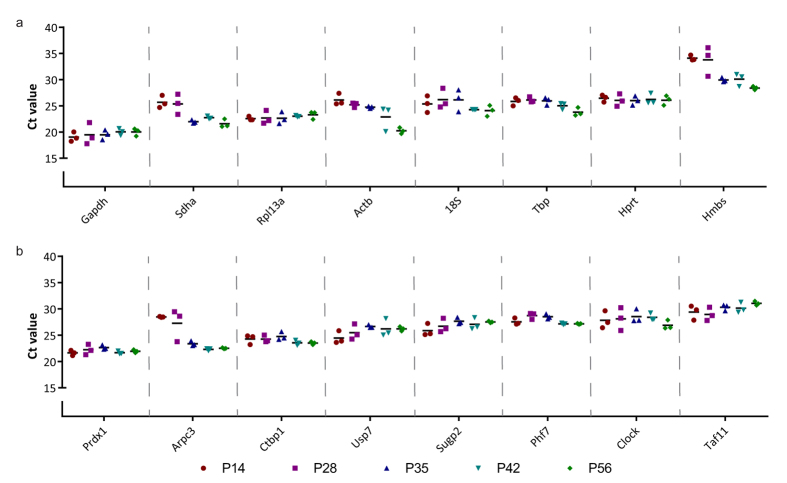
Ct values of reference genes for biological replicas of #4 mammary gland. Scatter plot depicting Ct values for all sixteen reference genes in the 4^th^ inguinal mammary gland over a developmental time series from pre-puberty (P14) to puberty (P28, P35 and P42) and adult (P56). Each data point represents a single biological replicate. (**a**) Traditionally used reference genes. (**b**) Novel candidate reference genes. Genes were plotted in order of their absolute expression levels, from high expression (lowest Ct value) to low expression (n = 3 biological replicates for each developmental time point).

To test the variation in expression levels of the traditional and novel candidate reference genes over a large developmental time course, we performed qRT-PCR analyses on cDNA synthesized from whole mouse mammary gland RNA preparations ranging from pre-puberty (P14) to early, mid- and late puberty (P28, P35 and P42) and adult virgin FVB mice (P56). When the expression levels (Ct values) of all sixteen (candidate) reference genes are compared, variable expression patterns across the different stages of development emerge for both the traditional (Fig. 3a) and the newly identified (Fig. 3b) genes. Some genes show a relatively stable pattern throughout, with all Ct values clustering closely together irrespective of the developmental time point. For instance, across pre-puberty and adulthood the Ct values for *Prdx1* and *Ctbp1* show a total spread of only 2.0 and 1.9 cycles, respectively. Other genes show more variation and are therefore probably less suitable as reference genes for our purpose. As an example, the expression of *Hmbs* and *Actb* shows a spread of 8.0 and 7.7 cycles, respectively. The 3^rd^ thoracic gland was analysed as a biological replicate and shows a similar pattern (Supplementary Fig. S1).

### GeNorm stability analysis of candidate reference genes

Reference genes can be ranked based on their expression stability over all samples using the GeNorm algorithm^12^. The underlying principle of this algorithm is that ideal reference genes show stable expression patterns regardless of the experimental parameters (in our case: different developmental time points). When multiple *bona-fide* reference genes are analysed, their average expression should best represent the “true” normalisation factor. Therefore, GeNorm not only scores for stable expression per candidate, but also for gene expression variation of each individual reference compared to the average reference gene expression. By performing a pairwise variation of any candidate reference gene compared to the average expression of the entire pool, a so-called M-value is calculated for each gene, which serves as a measure for gene stability: the lower the value, the more stably a gene is expressed. Next, the genes are ranked based on M-value, by performing stepwise exclusion of the gene with the highest M-value (i.e. poorest stability). M-value calculation and stepwise exclusion are repeated until only two genes remain. These final two genes are ranked based on their individual stability value.

**Figure 4:**
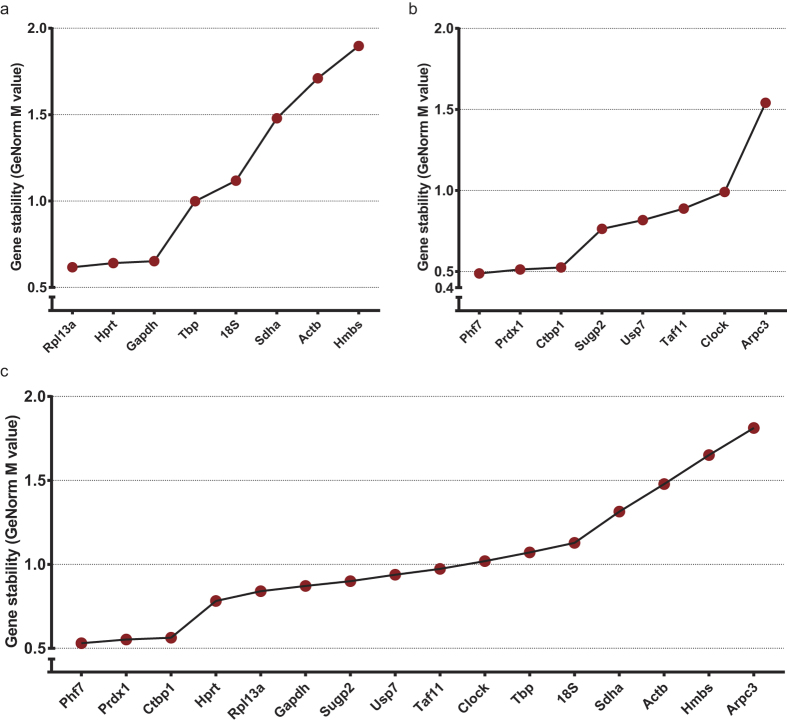
Reference gene ranking according to GeNorm. Graph showing candidate reference gene ranking according to the M-value for gene expression stability in the developing mammary gland. (**a**) Ranking of eight traditional reference genes. (**b**) Ranking of eight novel candidate reference genes. (**c**) Comprehensive ranking of the traditional and candidate reference gene sets. *Ctbp1, Phf7* and *Prdx1* have a lower M-value, indicating higher stability.

M-values should be as low as possible. However, they strongly depend on the complexity of the experimental samples and fixed criteria or cut offs do not exist^36^. For homologous samples, such as cell lines, the M-value can drop below 0.2. In contrast, for complex tissues M-values of 0.5 or even >1.0 have been reported^6^,^7^,^8^,^23^,^37^. When the traditional gene set was analysed, *Rpl13a, Hprt* and *Gapdh* came out as the best references, with an M-value of 0.62 for *Rpl13a* (Fig. 4a). GeNorm analysis for the novel candidates revealed *Phf7, Prdx1* and *Ctbp1* as best performing references in this set (Fig. 4b). The M-values for these genes (0.49, 0.51 and 0.53, respectively) are lower than the best M-value for the traditional gene set. Combined analysis of both novel (*Arpc3*, *Clock*, *Ctbp1*, *Phf7*, *Prdx1*, *Sugp2*, *Taf11* and *Usp7*) and traditional (*18S*, *Actb*, *Gapdh*, *Hbms*, *Hprt*, *Rpl13a*, *Sdha* and *Tbp*) reference genes confirms that *Phf7, Prdx1* and *Ctbp1* rank as the most stable genes across all developmental time points analysed, outperforming *Rpl13a, Hprt* and *Gapdh* (Fig. 4c).

### Comprehensive ranking of *Prdx1, Phf7* and *Ctbp1* as the most stable reference genes in the postnatal mammary gland

For a more comprehensive overview of reference gene suitability, we used the web-based tool RefFinder^26^ to rank the candidate reference genes according to three additional algorithms: DeltaCt^29^, BestKeeper^27^ and NormFinder^28^. DeltaCt ranks genes based only on the spread in Ct values (similar as depicted in Fig. 3), considering the gene with the least variation to be the best ranking reference. BestKeeper performs pairwise variations for all possible pairs of reference genes and calculates a geometric mean to determine which genes have highly similar expression profiles, creating a ranking based on the fit of the geometric means. NormFinder analyses the variance in expression data of all given candidate reference genes, with the assumption that an ideal reference gene should only differ mildly from the overall estimate.

The different approaches from the above mentioned algorithms lead to small differences in their respective reference gene rankings, but ideally the overall trend should be the same. Table 1 shows the comprehensive ranking of all novel candidates and traditional reference genes for the different algorithms. Indeed, the results confirm the GeNorm analysis: *Arpc3, Hmbs, Actb* and *Sdha* rank poorly for all algorithms and are thus considered to be less suitable reference genes in all tests. In contrast, *Prdx1, Phf7* and *Ctbp1* are ranked in the top four in all tests, complemented with either *Tbp*, *Hprt* or *Rpl13a* depending on the algorithm. A comprehensive ranking of the results from all four algorithms thus confirms *Prdx1, Phf7* and *Ctbp1* as the top three reference genes (Table 1 and Supplementary Fig. S2).

### Validation of novel reference genes by qRT-PCR analysis of *Wnt* gene expression

**Figure 5:**
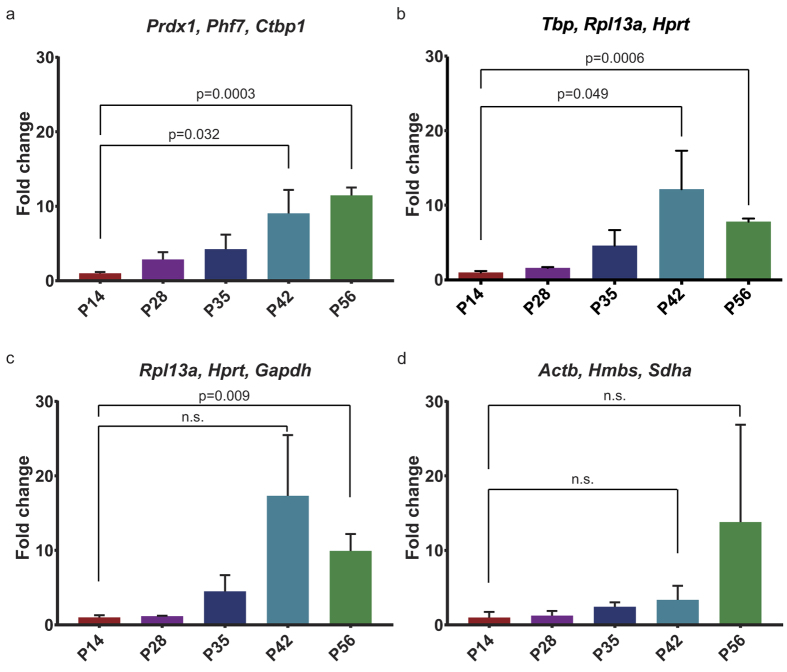
Reference gene validation. Bar graphs showing qRT-PCR analysis of *Wnt4* expression in the developing mouse mammary gland between P14 and P56. The lowest value (P14) was set to 1. (**a**) Relative *Wnt4* expression normalised to *Prdx1, Phf7* and *Ctbp1*, which were identified as novel reference genes in this study. (**b**) Relative *Wnt4* expression normalised to *Rpl13a, Hprt* and *Tbp*, which were identified as the best traditional reference genes in this study. (**c**) Relative *Wnt4* expression normalised to *Rpl13a, Hprt* and *Gapdh*, which were previously identified as the best reference genes for the mouse mammary gland^25^. (**c**) Relative *Wnt4* expression normalised to *Actb, Hmbs* and *Sdha*. Although frequently used as references, these genes rank poorly in our stability analyses. Statistically significant differences are indicated with their respective p-values. Error bars represent SEM (n = 3 biological replicates for each developmental time point). n.s. = not statistically significant.

To validate that *Prdx1, Phf7* and *Ctbp1* can be used as reliable reference genes for the postnatal mouse mammary gland, we tested their ability to reveal subtle changes in gene expression across mammary gland development. For this purpose, we focused on *Wnt* gene expression. Wnt signalling is crucial for mammary gland development and function, and Wnt-responsive stem cells reside in the mammary gland from an early time point on refs 38, 39, 40. Multiple *Wnt* genes have previously been shown to be expressed in the mammary gland, but so far, *Wnt* gene expression has mainly been studied using semi-quantitative methods^34^,^41^. Furthermore, *Wnt* genes are typically expressed at relatively low levels, making it challenging to detect expression level changes in RNA preparations from the whole mammary gland. *Wnt4* is known to be expressed in the adult mammary gland, where it plays a role in the estrus cycle^42^ and in side-branching during pregnancy^43^. A recent study also revealed a role for *Wnt4* in branching morphogenesis during puberty^44^, but if and how *Wnt4* expression levels change during postnatal mammary gland development remains incompletely understood. To this end, we quantified *Wnt4* expression across pre-puberty, puberty and adulthood. Only when *Prdx1, Phf7* and *Ctbp1* (our set of novel and preferred reference genes) are used for normalization, a steady increase in *Wnt4* levels is revealed between P14 and P56 (Fig. 5a). Subtle changes in *Wnt4* expression are also apparent when *Tbp*, *Rpl13a* and *Hprt* (the most reliable traditional reference genes) are used as a reference geneset (Fig. 5b). Using a set of even slightly less stable reference genes (*Rpl13a, Hprt* and *Gapdh*) already obscures this trend (Fig. 5c). Finally, when we normalized *Wnt4* expression to the three commonly used reference genes that were least stable in our analysis (*Actb, Hmbs* and *Sdha*), no statistically significant changes can be detected between the different developmental stages at all (Fig. 5d). This underscores the importance of using properly validated reference genes for the tissue of choice and confirms that the new reference gene set comprised of *Prdx1*, *Phf7* and *Ctbp1* allows small changes in developmental gene expression to be detected with high precision in a complex mammary tissue preparation.

## Discussion

Traditionally, reference genes used for normalization purposes in qRT-PCR experiments have been chosen based on their supposed role in cellular housekeeping processes. However, these genes do not perform well as reference genes in all settings, especially when complex tissues or larger developmental time series are concerned^33^. It is therefore important to critically select and evaluate candidate reference genes for each specific experimental condition.

In this study we report the identification and validation of novel reference genes for qRT-PCR analysis of a wide range of postnatal stages of mouse mammary gland development based on published mammary gland microarray expression data.

Efforts to detect novel reference genes for a diverse set of human samples by comparing large published datasets have been made before^14^,^15^,^16^,^17^,^18^. A study by de Jonge *et al*.^16^ analysed an astonishing number of 13.629 human microarray sets to determine which genes are stably expressed. The resulting genes, mostly encoding ribosomal proteins, showed improved stability values over traditionally used reference genes such as *GAPDH, ACTB* and *HPRT*. The top ranking genes for human samples also showed high stability values in mouse microarray data^16^. However, the resulting genes were not analysed for tissue-specificity and were not experimentally validated for the mouse mammary gland. Moreover, since almost all of the identified candidate reference genes were involved in very similar biological processes, they may be co-regulated, making it more difficult to test their suitability as true reference genes^12^.

Since reference gene stability can be particularly variable depending on the biological data set, reference genes should be validated on any organism- and tissue-specific experimental setting. We employed two separate microarray expression sets to obtain gene expression data for the mouse mammary gland at multiple developmental time points (puberty, pregnancy, lactation and involution) and for two different mouse strains^30^,^31^. We hypothesized that any gene showing stable expression across all these different conditions, would serve as a suitable reference gene for the postnatal mouse mammary gland. A total of 37 genes met our selection criteria (i.e. a logFC between −0.1 and 0.1 in four different cross comparisons). Of note, our approach allows the identification of both low, medium and highly expressed genes (Fig. 2f), unlike traditional “housekeeping” genes, which are often expressed at significantly higher levels than the experimental genes of interest. A final set of eight novel candidate reference genes was selected based on average expression levels, amplification efficiency and gene ontology analysis (Supplementary Table S1). The latter was performed to reduce the chance of selecting co-regulated genes with similar functions.

The resulting eight candidate reference genes were *Arpc3, Clock, Ctbp1, Phf7, Prdx1, Sugp2, Taf11* and *Usp7*. We compared the performance of these genes to eight traditional reference genes (*Actb, Gapdh, Hprt, Rpl13a, 18S, Hmbs, Sdha* and *Tbp*) using the GeNorm, DeltaCt, BestKeeper and NormFinder algorithms to determine the most stably expressed reference genes (Fig. 4 and Table 1)^12^,^27^,^28^,^29^. Out of the traditional reference gene set, *Tbp*, *Rpl13a* and *Hprt* perform better than the previously suggested combination of *Gapdh*, *Rpl13a* and *Hprt*^25^. However, our newly identified genes *Prdx1, Phf7* and *Ctbp1* rank as most stable overall. In addition, they are also in the top four of most stable reference genes in terms of their individual rankings (Table 1 and Supplementary Figure S2) and show a small spread in Ct values (Fig. 3), further supporting their suitability as reference genes in the mouse mammary gland. Interestingly, *CTBP1* and *PRDX1* have previously been found to be stably expressed in a variety of human samples, suggesting they may be more widely applicable^17^,^18^,^19^. In fact, *PRDX1* was recently shown to be the only reference gene commonly identified in fifteen published human reference gene studies^19^.

By using *bona-fide* and highly stable reference genes, even subtle changes in developmental gene expression can be picked up by qRT-PCR on a complex tissue sample (Fig. 5). Furthermore, in concordance with published guidelines for performing qRT-PCR experiments^36^,^45^ the use of three reference genes minimizes errors. Most qRT-PCR software programs accept the use of multiple reference genes for normalization. Alternatively, the geometric mean of three (or more) reference genes can be used for normalization, making this a feasible approach for most researchers today^46^. However, our validation experiment stresses the importance of selecting the most stable reference gene set (Fig. 5). In this respect, *Prdx1, Phf7* and *Ctbp1* perform better over a large developmental time series than previously identified reference genes for the mouse mammary gland^25^.

The fact that *Prdx1, Phf7* and *Ctbp1* outperform traditionally used reference genes, demonstrates the feasibility of our strategy to select tissue-specific, stably expressed genes based on existing microarray data. However, it should be noted that not all candidates showed stable expression upon further testing. In fact, *Arpc3* is one of the less well performing reference genes. One explanation is that our experimental samples contain an earlier developmental time point than the microarray datasets used to identify candidate genes. *Arpc3* expression in 2-week old mice (P14) deviates from the expression values at the other time points (Fig. 3b). Another explanation is that strain-specific differences could play a role. Candidates were identified based on microarray experiments on C57BL/6 and CD-1 tissues, but our experimental validation was performed on FVB tissues. This further underscores the importance of testing and validating candidate reference genes, even if they are tissue-specific.

In conclusion, large datasets such as microarray or RNAseq analyses allow the selection of tissue-specific reference genes, although these should always be tested experimentally for stability in the biological samples of interest. Using this approach, we have identified *Prdx1*, *Phf7* and *Ctbp1* as novel, tissue-specific reference genes for qRT-PCR studies of the postnatal mouse mammary gland, and validated that this reference gene set is suitable for detecting subtle gene expression changes that occur during the pre-pubertal, pubertal and adult stages.

## Materials and Methods

### Animals

FVB/N mice were purchased from Envigo. Breeding for the appropriate developmental time points (P14, P28, P35, P42 and P56) was performed in-house. Mice were housed in open conventional cages on a 12 h light/dark cycle and received food and water *ad libitum*. All experiments were performed in accordance with institutional and national guidelines and regulations. All experimental protocols were approved by the Animal Welfare Committee of the University of Amsterdam. As biological replicates, n = 3 animals were used for each developmental time point.

### Selection of candidate reference genes

A total of eight reference genes commonly used in qRT-PCR analyses for mouse tissues for which primers were already available were selected as “traditional” reference genes. Another set of eight novel candidate reference genes was selected from published mammary gland microarray datasets (GEO accession numbers GSE5831^30^ and GSE6453^31^) as described in the text. Briefly, GEO2R analysis was performed in RStudio using the GEOquery^32^ and limma^47^ R packages from the Bioconductor project to determine the log fold change (logFC) between virgin and lactating mice (GSE5831), pregnant and involuting mice (GSE5831), 3 versus 7 week old mice (GSE6453) and 4 versus 6 week old mice (GSE6453).

Genes with a logFC > −0.1 | <0.1 in each of these comparisons were selected as putative tissue-specific reference genes. These gene lists are provided in Supplementary Table S1. Gene ontology term analysis was performed using the “biological process” and “molecular function” GO terms from the GEO2R analysis to ensure genes with different cellular functions were used to build the final set of eight candidate reference genes to prevent the selection of co-regulated genes.

### Primer design and validation

Primer sequences for the eight traditional reference genes were already available in the lab. Novel primers were selected from Primerbank (https://pga.mgh.harvard.edu/primerbank/) when available or designed with the Roche Universal Probe Library Assay Design Centre (https://lifescience.roche.com/). Only intron-spanning primer pairs were used. Specific amplification was confirmed by a single peak after melting curve analysis, a single band of appropriate size on an agarose gel and sequence verification. The amplification efficiency of each primer pair was determined by generating standard curves, using different dilutions of mixed mammary gland cDNA samples as input (see below). The amplification efficiency of the primers was calculated from the slope of the standard curve using Biogazelle qBase + software. All primer sequences, amplicon lengths and amplification efficiencies are available in Supplementary Table S2.

### RNA isolation and cDNA synthesis

Total RNA was isolated from the pooled left and right #4 (inguinal) mammary glands of each biological replicate (n = 3 for each time point) using TRIzol reagent (Fisher Scientific) according to the manufacturer’s guidelines. RNA from the pooled left and right #3 mammary glands was isolated separately. Residual genomic DNA was digested by RQ1 RNAse-free DNAse treatment (Promega) according to the manufacturer’s instructions. The RNA concentration was determined using a Nanodrop spectrophotometer. The purity of all samples was assessed by the absorbance ratios of OD260/280 and OD260/230. cDNA was synthesized from 2 μg RNA using SuperScript^®^ II Reverse Transcriptase (Invitrogen) and Random Hexamers (Fisher Scientific), according to the manufacturer’s instructions. The cDNA was diluted 10-fold for subsequent qRT-PCR analysis. Standard curves were generated by diluting the cDNA 2-, 5-, 10-, 100- and 1000-fold.

### Quantitative Real-time PCR (qRT-PCR)

qRT-PCR was performed using an Applied Biosystems 7500 Real-Time PCR System. PCR reactions (total 20 μl) were set up containing 13 μl RNAse-free H_2_O, 4 μl 5× HOT FIREPol^®^ EvaGreen^®^ qRT-PCR Mix Plus ROX (Solis Biodyne), 0.5 μl of each specific forward and reverse primer (10 μM stock) and 2 μl of diluted cDNA template. The reactions were set up in technical triplicates in 96-well PCR plates. One negative control (no-RT) reaction was included for each sample/primer combination. Thermal cycling was performed, starting with an initial step at 95 °C for 10 min, followed by 40 cycles of denaturation at 95 °C for 15 s and annealing at 60 °C for 60 s. Each run was completed with a melting curve analysis.

### Testing expression stability of candidate reference genes

Raw Ct values were used for GeNorm analysis using the Biogazelle qBase + software^12^. All technical triplicates were included to calculate a single Ct value for each gene and biological sample. To minimize the effect of spread within technical replicates, Ct values were calculated based on the median^48^. Expression stability values (M) were calculated as described in the text.

Median Ct values of the technical triplicates were also used as import for the web-based tool RefFinder^26^ (http://fulxie.0fees.us). The resulting rankings for the DeltaCt, BestKeeper and NormFinder algorithms were used to assign an appropriate weight to each of the individual reference genes. To this end, all reference genes were given a value (1–16) for their position. The same was done for the GeNorm ranking derived from the qBase + software. An overall final ranking was calculated based on the geometric mean of their four individual positions.

### *Wnt4* expression analysis

Relative expression levels of *Wnt4* were calculated by the comparative Delta-Ct method^49^,^50^, taking the different amplification efficiencies into account, and were normalized to three reference genes (either *Prdx1*, *Phf7* and *Ctbp1; or Rpl13a, Hprt* and *Tbp;* or *Rpl13a, Hprt* and *Gapdh;* or *Actb, Hmbs* and *Sdha*) using the Biogazelle qBase + software. Statistical analyses were done using qBase + Statistics Wizard and the Analysis Toolpack Plugin for Microsoft Excel 2016. An F-test for variances was performed to test whether the data distribution was equal. Subsequently, a t-test was performed to test whether there was a difference between respective samples.

## Additional Information

**How to cite this article**: van de Moosdijk, A. A. A. and van Amerongen, R. Identification of reliable reference genes for qRT-PCR studies of the developing mouse mammary gland. *Sci. Rep.* **6**, 35595; doi: 10.1038/srep35595 (2016).

## Supplementary Material

Supplementary Information

## Figures and Tables

**Table 1:** Reference gene ranking according to four algorithms.

Ranking	1	2	3	4	5	6	7	8	9	10	11	12	13	14	15	16
Delta CT	Prdx1	Ctbp1	Phf 7	Tbp	Hprt	Rpl13a	Sugp2	Gapdh	Clock	18S	Usp7	Taf11	Sdha	Actb	Hmbs	Arpc3
BestKeeper	Prdx1	Ctbp1	Rpl13a	Phf 7	Tbp	Hprt	Sugp2	Gapdh	Taf11	Usp7	Clock	18S	Sdha	Actb	Hmbs	Arpc3
NormFinder	Phf 7	Tbp	Prdx1	Ctbp1	Hprt	Rpl13a	Clock	Sugp2	Gapdh	18S	Usp7	Taf11	Sdha	Actb	Hmbs	Arpc3
GeNorm	Phf 7	Prdx1	Ctbp1	Hprt	Rpl13a	Gapdh	Sugp2	Usp7	Taf11	Clock	Tbp	18S	Sdha	Actb	Hmbs	Arpc3
Overall ranking	Prdx1	Phf 7	Ctbp1	Tbp	Rpl13a	Hprt	Sugp2	Gapdh	Clock	Usp7	Taf11	18S	Sdha	Actb	Hmbs	Arpc3

DeltaCt, BestKeeper, NormFinder and GeNorm were used to rank reference genes for suitability. Ranking was performed in RefFinder (DeltaCt, BestKeeper and NormFinder) or qBase + (GeNorm). Individual rankings are shown in the top four rows. The overall comprehensive ranking is shown in the bottom row. A similar analysis performed separately on the traditional and the novel reference gene sets is presented in Supplementary Fig. S2.
