# Supplementary material for: Identification of reliable reference genes for qRT-PCR studies of the developing mouse mammary gland
Source: Sci Rep. 2016 Oct 18;6:35595. doi: 10.1038/srep35595 (PMC5067587; doi:10.1038/srep35595)
Supplement: Supplementary Information [file srep35595-s1.pdf]

Identification of reliable reference genes for qRT-PCR studies of the developing mouse mammary gland

Anoeska Agatha Alida van de Moosdijk and Renée van Amerongen

Supplementary Files

- Supplementary Figure S1 (this PDF)
- Supplementary Figure S2 (this PDF)
- Supplementary Table S1 (uploaded separately as an .xlsx file)
- Supplementary Table S2 (this PDF)

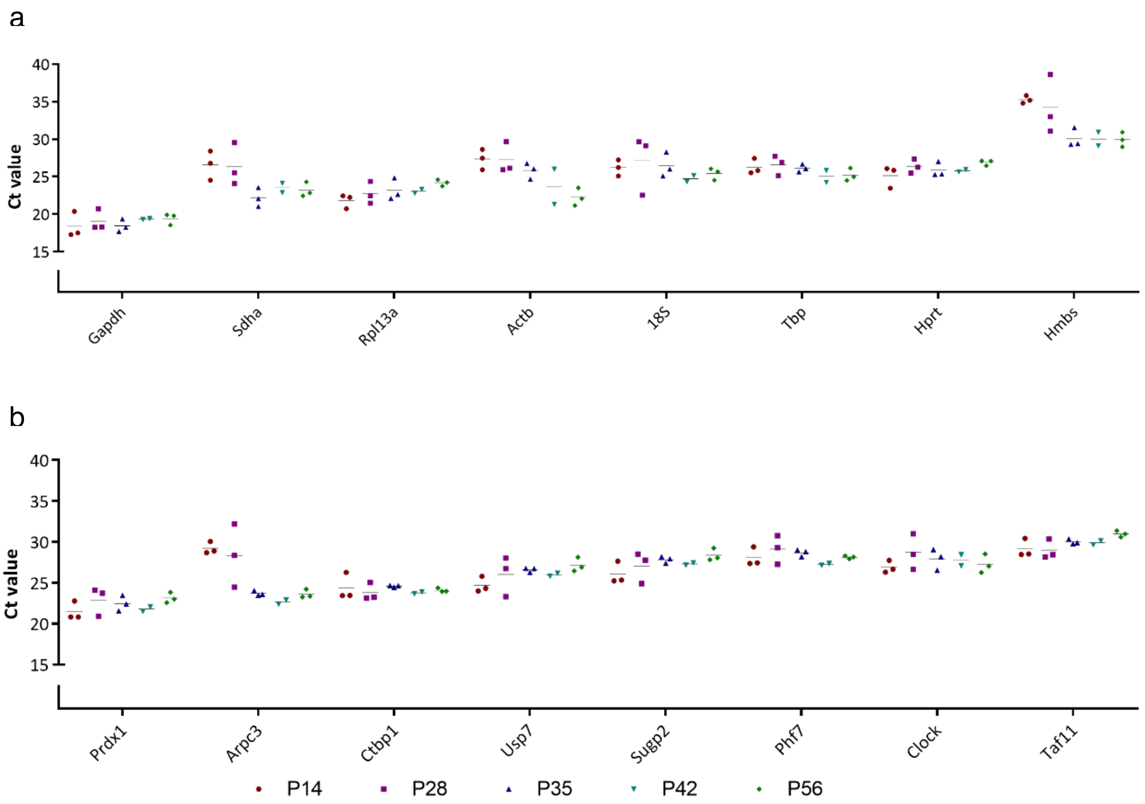

Supplementary figure S1. **Ct values and reference gene ranking for #3 mammary gland**. Scatter plot depicting spread in expression of candidate reference genes in different stages of the #3 mouse mammary gland. a) Traditional reference genes b) Novel candidate reference genes.

van de Moosdijk et al., Supplementary Figure S2

(A)

Ranking Order (Better--Good--Average)								
Method	1	2	3	4	5	6	7	8
Delta CT	Tbp	Rpl13a	Hprt	18S	Gapdh	Sdha	Actb	Hmbs
BestKeeper	Rpl13a	Tbp	Hprt	Gapdh	18S	Sdha	Actb	Hmbs
Normfinder	Tbp	Rpl13a	Hprt	18S	Sdha	Gapdh	Actb	Hmbs
GeNorm qBase	Rpl13a	Hprt	Gapdh	Tbp	18S	Sdha	Actb	Hmbs
Recommended comprehensive ranking	**Rpl13a**	**Tbp**	**Hprt**	**Gapdh**	**18S**	**Sdha**	**Actb**	**Hmbs**

(B)

Ranking Order (Better--Good--Average)								
Method	1	2	3	4	5	6	7	8
Delta CT	Prdx1	Sugp2	Ctbp1	Phf7	Usp7	Taf11	Clock	Arpc3
BestKeeper	Prdx1	Ctbp1	Phf7	Sugp2	Taf11	Usp7	Clock	Arpc3
Normfinder	Prdx1	Phf7	Ctbp1	Sugp2	Clock	Usp7	Taf11	Arpc3
GeNorm qBase	Phf7	Prdx1	Ctbp1	Sugp2	Usp7	Taf11	Clock	Arpc3
Recommended comprehensive ranking	**Prdx1**	**Phf7**	**Ctbp1**	**Sugp2**	**Usp7**	**Taf11**	**Clock**	**Arpc3**

Supplementary Figure S2.
Comprehensive ranking of the old/traditional (A) and novel candidate reference genes (B) using RefFinder (available at http://fulxie.0fees.us/?type=reference). A combined comprehensive ranking of both the traditional and novel candidate genes is presented in Table 1.

Gene name	Accession Number	Primer sequence (5' – 3')	Amplicon length	PCR Efficiency %
**Reference genes**				
18S	NR_003278	F: CCGCCGCCATGTCTCTAGT<br>R: CTTTCCTCAACACCACATGAGC	150 bp	90
Arpc3	NM_019824	F: TGAAGCGGACAGGACATTGAT<br>R: AGTTGGTGATTCCTAGCGTGT	122 bp	92
Actb	NM_007393	F: GAGCTGCCTGACGGCCAGGT<br>R: TACTCCTGCTTGCTGATCCA	365 bp	72
Clock	NM_001289826	F: TTTACAGGCGTTGTTGATTGGA<br>R: ACGCAAGGCCGTCTTCTG	92 bp	97
Ctbp1	NM_001198859	F: GTGCCCTGATGTACCATACCA<br>R: GCCAATTCGGACGATGATTCTA	83 bp	92
Gapdh	NM_001289726	F: CTGGTGCTGCCAAGGCT<br>R: CTGCTTCACCACCTTCTTGATGTCATCATA	161 bp	99
Hmbs	NM_013551	F: GCCCCATGTGCCTTCAGTC<br>R: TCTTTGAGCCGTTTTCTTCCGC	151 bp	101
Hprt	NM_013556	F: TGTTGTTGGATATGCCCTTG<br>R: TTGCGCTCATCTTAGGCTTT	108 bp	97
Phf7	NM_027949	F: TGTTTGCCTTCTATGCCTTCAA<br>R: GGCAGTCTGCTAGACAGGATAAG	114 bp	88
Prdx1	NM_011034	F: AATGCAAAAATTGGGTATCCTGC<br>R: CGTGGGACACACAAAAGTAAAGT	150 bp	96
Rpl13a	NM_009438	F: CCCTCCACCCTATGACAAGA<br>R: GCCCCAGGTAAGCAAACTT	95 bp	101
Sdha	NM_023281	F: GTTGCTGTGTGGCTGATCG<br>R: GCACAGTGCAATGACACCAC	149 bp	89
Sugp2	NM_001168290	F: TGACCAGAGGCCGATCTCTAA<br>R: GGAAGTAATACGACTGGCAACAG	158 bp	105
Tbp	NM_013684	F: GTCATTTTCTCCGCAGTGCC<br>R: GCTGTTGTTCTGGTCCATGAT	151 bp	94
Taf11	NM_026836	F: GATGGAGACGCGGACTTGAAA<br>R: GCAGGAGTAAGTAAGGACGAGTT	113 bp	99
Usp7	NM_001003918	F: CCACAAGGAAAACGACTGGG<br>R: GTAACACGTTGCTCCCTGATT	196 bp	88
**Target genes**				
Wnt4	NM_009523	F: ACTGGACTCCCTCCCTGTCT<br>R: TGCCCTTGTCACTGCAAA	109 bp	97

Supplementary table S2. **Overview of all genes and primer sequences used in this study.** The length of the amplicon and the efficiency of the primers is also listed.
